# Managing chronic wounds during novel coronavirus pneumonia outbreak

**DOI:** 10.1093/burnst/tkaa016

**Published:** 2020-04-07

**Authors:** Rui Wang, Yanzhen Peng, Yufeng Jiang, Jianwen Gu

**Affiliations:** 1 Wound Healing Department, PLA Strategic Support Force Characteristic Medical Center, Beijing 100101, China; 2 Center of Minimally Invasive Gynecological Surgery of Beijing Obstetrics and Gynecology Hospital Affiliated to Capital Medical University, Beijing 10006, China; 3 Prevention and Control Group of COVID-19, PLA Strategic Support Force Characteristic Medical Center, Beijing 100101, China

**Keywords:** managing chronic wounds, novel coronavirus pneumonia

## To the Editor

In December of 2019, a widespread outbreak of novel coronavirus pneumonia (NCP) occurred in Wuhan, China. On January 12, 2020, the virus causing NCP was named as “2019-nCoV” by the World Health Organization [1,2]. The World Health Organization renamed NCP as “COVID-19”. COVID-19 is a highly contagious respiratory virus that poses a serious threat to human health worldwide. At present, COVID-19 has been classified as a Class B infectious disease in China, and management of Class A infectious diseases has been adopted. As of March 10, 2020, 80 932 cases were diagnosed and confirmed as COVID-19 in China, with 60 002 cured cases and 3140 death. Meanwhile, 34 656 cases were diagnosed and confirmed as COVID-19 abroad, in which 4188 cases were cured and 968 cases died. Therefore, the prevention and control of pandemic COVID-19 enters a critical period.

The sudden outbreak of COVID-19 makes the management of chronic wounds more difficult. Among the chronic wound patients recorded from January 2018 to January 2019, the number of patients over 50 years old accounted for 76.74%, of which 78.25% were complicated with underlying diseases [3]. Among the underlying diseases of chronic wound patients, the top four diseases were diabetes, cardiovascular and cerebrovascular diseases, hypertension and respiratory diseases. The basic diseases and older age are the susceptibility factors of the COVID-19, as announced by the National Health Commission. Therefore, the conflict between the need for managing wound and the risk of suffering communicable disease during the prevention and control of COVID-19 appears to be a particular dilemma for patients with chronic wounds.

**Figure 1.**
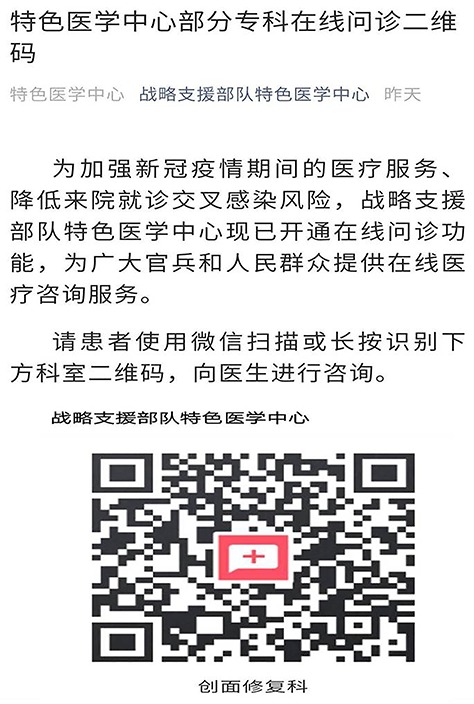
WeChat online consultation was established officially in PLA Strategic Support Force Characteristic Medical Center. Quick response (QR) code was created for online consultation of some specialties. Translation of the figure: In order to provide the medical services during the novel coronavirus epidemic and reduce the risk of cross infection, the online service is able to provide instant medical consultation for patients. Please scan the QR code and identify the department to consult the respective doctors

**Figure 2.**
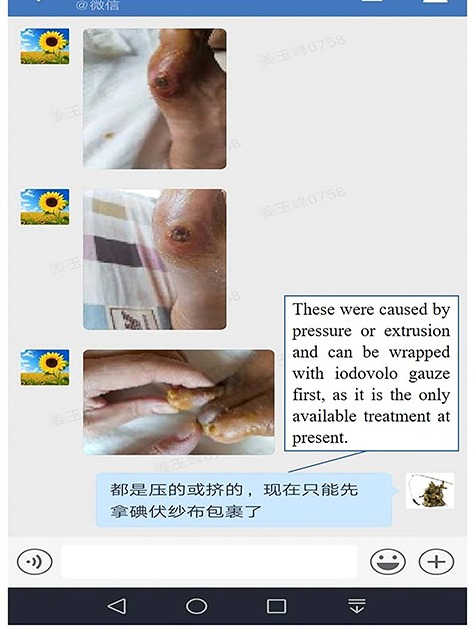
Screenshot of WeChat conversation between patients and doctors. Instructing homebound patient in basic skill of managing wounds on WeChat

**Figure 3.**
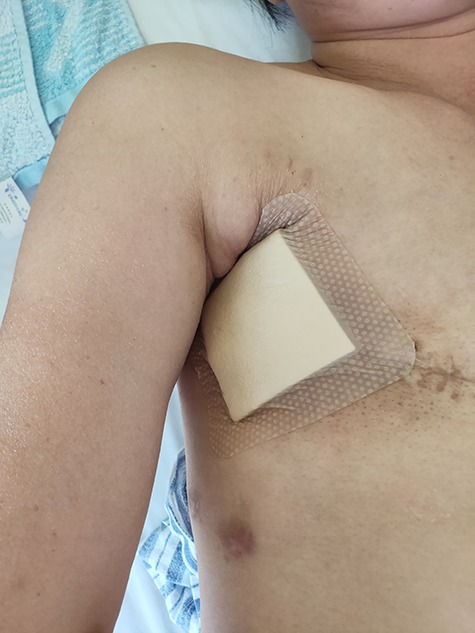
Foam dressing covering wound

**Figure 4.**
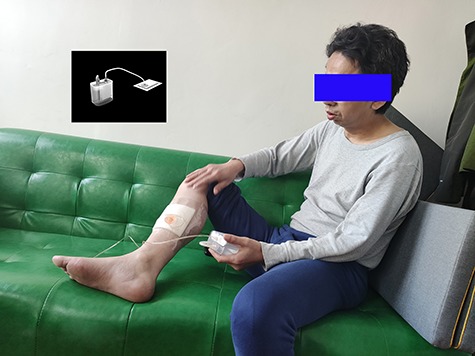
Instructing homebound patient in facilitating negative pressure wound therapy on WeChat

**Figure 5.**
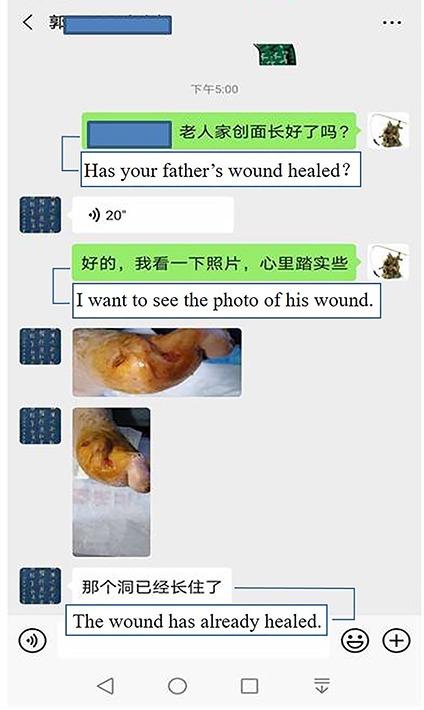
Screenshot of WeChat conversation between patients and doctors. Wound nearly healed by remote management

According to the guidelines and consensus on epidemic prevention and control recently issued by the National Health Commission and relevant agencies [4–7], and telemedicine being able to reduce travel time for patients and medical staff [8], we have established a management strategy for patients with chronic wounds outside of the hospital. We have some experiences and advice as following:

1. To minimize the exposure risk of patients during the epidemic, we take advantage of a comprehensive online communication strategy and encourage patients to consult their doctors about wound-condition by WeChat, which was used officially in our hospital (Figs.1, 2). WeChat is a messaging and calling application that allows people to easily connect with others. It's the all-in-one communications app for text (SMS/MMS), voice and video calls, and files. It also supports communication for group talk. It can be used in most smartphones and consumes a small amount of net resource. It is frequentlly used in China, as FaceBook, Skype and LINE Apps used by American, South Korean and Japanese. Through the wound pictures uploaded by the patient, or communication with medical staffs, the doctor can give the preliminary advice.
2. We have arranged for doctors to manage the wounds of the patients, who were treated in our department and left hospital before their wounds healed completely because of prevention and control of COVID-19. We tried to avoid secondary damage such as amputation because of lacking active treatment of the diabetic foot in this way.
3. We have taken advantage of modern dressing such as foam dressings and negative pressure wound therapy (Figs. 3,4), for dressing change so as to prolong the intervals and decrease frequencies of wound dressing change.
4. We also instruct these patients to master some basic wound managing skills in a short period, so that they can change their own dressing for some uncomplicated wound at home.
5. If the patient needs some treatments such as debridement, operation, or revascularization because of infection, necrosis and/or gangrene, our team member will online consult some specialists in different fields according to the patient's condition. As such, the patient will get the appropriate advice online, instead of visiting the doctors. If the patient does need hospitalization, the nearest professional wound-healing clinic is the best choice, and fever and novel coronavirus pneumonia screen should be performed in fever clinic before admission.
6. Up to March 6, 2020, we managed 15 patients’ wounds in one month. None of their wound bed were getting worse and out of control. Six patients’ wounds have been healed (Fig. 5). Other 9 patients’ are getting improved as well.

**Figure 6.**
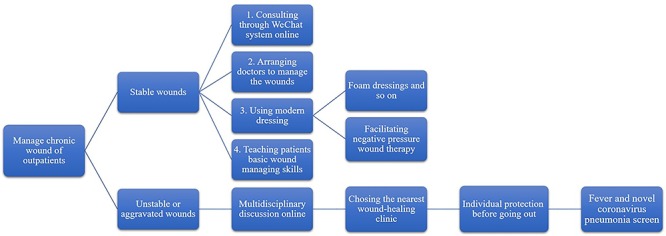
Flow chart of managing chronic wounds during novel coronavirus pneumonia outbreak

In summary, it is an ideal way to manage chronic wound by modern dressing, facilitating technology such as negative pressure wound therapy and tele-medicine to maintain effective therapy as the flow chart (Fig. 6), and meanwhile to avoid the exposure risk of COVID-19 during the critical COVID-19 pandemic prevention and control period. This managing strategy would also be applied in treating patients in earthquake, plague, and other inconvenient situation.

## Prior Presentation

The authors declare that this paper has not been published previously.
